# Extracellular Vesicle Surface Signatures in IPF Patients: A Multiplex Bead-Based Flow Cytometry Approach

**DOI:** 10.3390/cells10051045

**Published:** 2021-04-28

**Authors:** Miriana d’Alessandro, Piera Soccio, Laura Bergantini, Paolo Cameli, Giulia Scioscia, Maria Pia Foschino Barbaro, Donato Lacedonia, Elena Bargagli

**Affiliations:** 1 Respiratory Diseases Unit, Department of Medical and Surgical Sciences & Neurosciences, Siena University Hospital, 53100 Siena, Italy; bergantini@student.unisi.it (L.B.); cameli3@student.unisi.it (P.C.); bargagli2@unisi.it (E.B.); 2 Department of Medical and Surgical Sciences, University of Foggia, 71122 Foggia, Italy; piera.soccio@unifg.it (P.S.); giulia.scioscia@unifg.it (G.S.); mariapia.foschino@unifg.it (M.P.F.B.); donato.lacedonia@unifg.it (D.L.); 3 Institute of Respiratory Diseases, Policlinico Riuniti of Foggia, 71122 Foggia, Italy

**Keywords:** extracellular vesicles, IPF, biomarkers, prognosis

## Abstract

Background: Extracellular vesicles (EVs) are secreted by cells from their membrane within circulation and body fluids. Knowledge of the involvement of EVs in pathogenesis of lung diseases is increasing. The present study aimed to evaluate the expression of exosomal surface epitopes in a cohort of idiopathic pulmonary fibrosis (IPF) patients followed in two Italian Referral Centres for Interstitial Lung Diseases, comparing them with a group of healthy volunteers. Materials and Methods: Ninety IPF patients (median age and interquartile range (IQR) 71 (66–75) years; 69 males) were selected retrospectively. Blood samples were obtained from patients before starting antifibrotic therapy. A MACSPlex Exosome Kit, human, (Miltenyi Biotec, Bergisch-Gladbach, Germany), to detect 37 exosomal surface epitopes, was used. Results: CD19, CD69, CD8, and CD86 were significantly higher in IPF patients than in controls (*p* = 0.0023, *p* = 0.0471, *p* = 0.0082, and *p* = 0.0143, respectively). CD42a was lower in IPF subjects than in controls (*p* = 0.0153), while CD209, Cd133/1, MCSP, and ROR1 were higher in IPF patients than in controls (*p* = 0.0007, *p* = 0.0050, *p* = 0.0139, and *p* = 0.0335, respectively). Kaplan-Meier survival analysis for IPF patients: for median values and a cut-off of 0.48 for CD25, the two subgroups showed a significant difference in survival rate (*p* = 0.0243, hazard ratio: 0.52 (95%CI 0.29–0.92); the same was true for CD8 (cut-off 1.53, *p* = 0.0309, hazard ratio: 1.39 (95%CI 0.75–2.53). Conclusion: Our multicenter study showed for the first time the expression of surface epitopes on EVs from IPF patients, providing interesting data on the communication signatures/exosomal profile in serum from IPF patients and new insights into the pathogenesis of the disease and a promising reliability in predicting mid-term survival of IPF patients.

## 1. Introduction

Extracellular vesicles (EVs) are secreted by the cell membrane into the circulation and body fluids. They contain cytoplasmic components, such as proteins and miRNA. Since EVs express surface proteins linked to their origin, they can adhere and fuse with circulating or distant resident cells. This capturing property of distant cells enables EVs to shuttle functional nucleotides or proteins, providing horizontal transfer of a variety of biological molecules [1]. EVs include exosomes, previously distinguished on the basis of size (40–150 nm) [2]. Exosomes are EVs generated by inward budding of the cell membrane (endocytosis), subsequent shaping of multivesicular bodies, and release by exocytosis [3]. During release into the extracellular space, the peripheral membrane of the multivesicular bodies fuses and the exosomes incorporate various membrane proteins, including tetraspanins (CD9, CD63, CD81). These surface proteins are recognized as markers of exosomes. Due to overlapping sizes and a lack of specific markers for each EV component, the International Society for Extracellular Vesicles recommends using an umbrella term, EVs, to describe all vesicle types [2,4].

In respiratory medicine, there is increasing evidence of the involvement of EVs in the pathogenesis of lung diseases including lung cancer, chronic obstructive pulmonary disease, and pulmonary fibrosis [4,5,6].

In the field of lung cancer, studies on EVs are extensively growing, especially about the potential of EVs as diagnostic biomarkers of lung cancer [6,7]. Exosomes have been demonstrated to modify the tumor microenvironment, promoting metastasis and angiogenesis and modulating immune responses. EVs can regulate cancer-associated fibroblasts.

A few reports have investigated the role of miRNA in a murine bleomycin-induced lung fibrosis model and in IPF patients. Their results suggest that miRNAs could be useful prognostic indicators of disease [4].

Unlike conventional biomarkers of IPF, such as Krebs von den Lungen-6 (KL-6) [8,9,10,11,12,13] and matrix metalloproteases, EVs and miRNAs may be quite specific for cell conditions in pulmonary fibrosis. Alone or combined with conventional biomarkers, they may offer insights into the pathogenesis and progression of IPF.

The aim of the present study was to compare the expression of exosomal surface epitopes in a cohort of IPF patients monitored in two Italian referral centres for interstitial lung diseases (ILDs) and in a control group of healthy volunteers.

## 2. Materials and Methods

### 2.1. Study Population

Ninety IPF patients (median age and interquartile range (IQR) 71 (66–75) years; 69 males), monitored at the Siena Regional Referral Centre for Interstitial Lung Diseases and Foggia Regional Referral Centre for Rare Pulmonary Diseases, were selected retrospectively. Patients with clinical evidence of concomitant infections, malignancies, and/or acute exacerbations of the disease were excluded, as well as IPF patients with a radiological pattern of indeterminate usual interstitial pneumonia (UIP). The diagnosis of IPF was confirmed by multidisciplinary discussion according to international guidelines [14]. Only fifteen patients (16.6%) had histological confirmation of IPF. As control group, we enrolled 19 healthy volunteers (median age (IQR) 63 years (52–65); 12 males). Data were retrospectively collected from medical reports and entered in an electronical database by physicians experienced in the management of ILD [15].

Blood samples were obtained from patients before starting antifibrotic therapy [16,17]. Lung function tests and high-resolution computed tomography (HRCT) of the chest were performed for all patients for diagnostic purposes. They were performed according to American Thoracic Society/European Respiratory Society (ATS/ERS) standards [18] using a Jaeger body plethysmograph with correction for temperature and barometric pressure.

Demographic and clinical data, comorbidities, family history, lung function parameters, including diffusion capacity of carbon monoxide (DLCO), and radiological features were obtained from the medical records and entered in an electronic database for statistical analysis.

**Figure 1:**
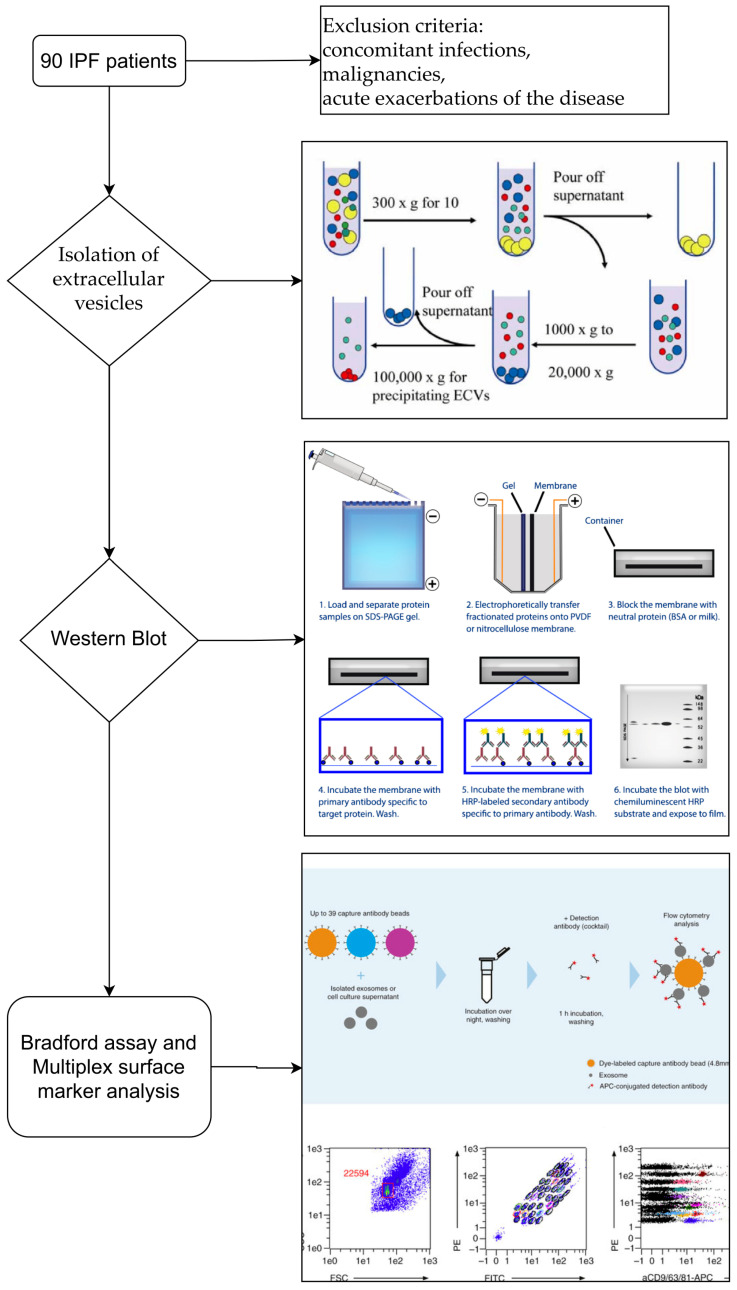
Flowchart of the study design. The description of the study design from patient selection to characterization of extracellular vesicles.

The description of the study design with a flow chart is reported in Figure 1.

Healthy controls and patients gave their written informed consent to participate in the study, which was approved by our local ethics committee (C.E.A.V.S.E., Tuscany, Italy, Markerlung number 17431).

### 2.2. Human Blood Samples

Serum samples were processed as follows: 6 mL blood samples from IPF patients and healthy volunteers were isolated in serum-separating tubes. The blood was spun at 2500× *g* for 10 min. Samples were then frozen at −80 °C.

### 2.3. Isolation of Extracellular Vesicles

According to the manufacturer’s instructions [19], serum was diluted with an equal volume of PBS. Cells and cell debris were removed by serial centrifugations at 2000× *g* for 30 min and 10,000× *g* for 45 min at 4 °C. The pellets were resuspended and filtered through a 0.22 μm membrane. EVs were isolated by ultracentrifuging the supernatant at 100,000× *g* for 2 h at 4 °C. The EVs were resuspended in 100 μL PBS and stored at −80 °C.

### 2.4. Western Blot

The exosome protein concentration was measured by the Bradford protein assay (Biorad, Hercules, CA, USA). Equivalent amounts of protein (80 µg) from exosomes were separated by SDS-polyacrylamide 12% gel and transferred to PVDF (Biorad). The membrane was blocked with 5% milk in Tris-buffered saline with 0.1% Tween-20 and incubated overnight at 4 °C with primary antibodies: CD81 (#10630D, Invitrogen, Carlsbad, CA, USA), Alix (#SC53540, Santa Cruz Biotechnology, Dallas, TX, USA), and β-actin (#A5441, Sigma-Aldrich, St. Louis, MO, USA) in blocking solution. After three washes with Tris-buffered saline/0.1% Tween-20, the membrane was incubated for 1 h with HRP-conjugated secondary antibodies. Immunoreactive bands were detected with ECL substrate (Biorad, Hercules, CA, USA).

### 2.5. Bradford Assay

The Bradford protein assay was used to measure protein concentrations in BAL fluid samples, as previously reported [20,21,22]. The principle of the assay is the binding of protein molecules to Coomassie dye under acidic conditions which results in a colour change from brown to blue. This method measures the basic amino acid residues arginine, lysine, and histidine, which contribute to the formation of the protein-dye complex. EV samples were processed in a 96-well plate assay using Bradford reagent for 0.1–1.4 mg/mL protein (Sigma-Aldrich, St. Louis, MO, USA). Standard curves were obtained using bovine serum albumin (BSA) (Sigma Aldrich, St. Louis, MO, USA) according to the manufacturer’s instructions. The samples were incubated for 20 min, and fluorescence was read at 595 nm with a Perkin Elmer Victor X4 fluorimeter. EV proteins were expressed in mg/mL.

### 2.6. Multiplex Surface Marker Analysis

MACSPlex analysis was performed using the MACSPlex Exosome Kit, human (Miltenyi Biotec, Bergisch-Gladbach, Germany), which detects 37 exosomal surface epitopes plus two isotype controls [19]. After indirect determination of the EV concentration by quantifying the protein concentration, EV-containing samples were processed as follows: isolated EVs (4–20 μg protein) from each sample were diluted with MACSPlex buffer (MPB) to a final volume of 120 μL and loaded into 1.5-mL tubes with 15 μL MACSPlex Exosome Capture Beads. After overnight incubation at room temperature protected from light in an orbital shaker (450 rpm), 500 μL MACSPlex Buffer was added to each tube and centrifuged at room temperature at 3000× *g* for 5 min. The supernatant was aspirated and 5 μL MACSPlex Exosome Detection Reagent CD9, CD63, and CD81 were added to each tube followed by incubation for 1 h at room temperature protected from light in an orbital shaker (450 rpm). The washing step was repeated, and 500 μL MACSPlex Buffer was added to each tube followed by incubation for 15 min at room temperature protected from light in an orbital shaker (450 rpm). EV-containing samples were centrifuged and the supernatant aspirated, leaving about 150 μL in the tube. Flow cytometric analysis was carried out on a Facs CantoII flow cytometer followed by Kaluza Analysis 2.1 (Beckman and Coulter Life Sciences, CA, USA) (Figure S1a,b). For further analysis, background values of the control sample (PBS) of each run were subtracted from the sample values. Exosomal surface epitope concentrations were obtained from the ratio of beads + EVs + Ab to the corresponding controls (capture beads + Ab). Surface marker concentrations below that of the corresponding control antibody included in the kit, taken as a measurement threshold, were regarded as negative.

### 2.7. Statistical Analysis

The results were expressed as means and standard deviations (SD) or medians and quartiles (25th and 75th percentiles) for continuous variables, as appropriate. One-way ANOVA non-parametric test (Kruskal-Wallis test) and Dunn test were performed for multiple comparisons. The Chi-squared test was used for categorical variables. The Kaplan-Meier estimator test was used for survival analysis, and the hazard ratio (Mantel-Haenszel) was calculated. A *p* value less than 0.05 was considered statistically significant. Statistical analysis was performed by GraphPad Prism 9 software.

## 3. Results

Clinical, demographic, and immunological data are reported in Table 1. As expected, most of our patients were males (77%) and current/former smokers (53%). No significant differences in age, gender or smoking history were observed between the IPF group and controls. On average, functional assessment at baseline showed mild restrictive impairment of lung volumes, associated with a moderate reduction in DLCO percentages. Fifty-three patients started antifibrotic therapy with pirfenidone (59%), while 37 patients (41%) were treated with Nintedanib.

**Figure 2:**
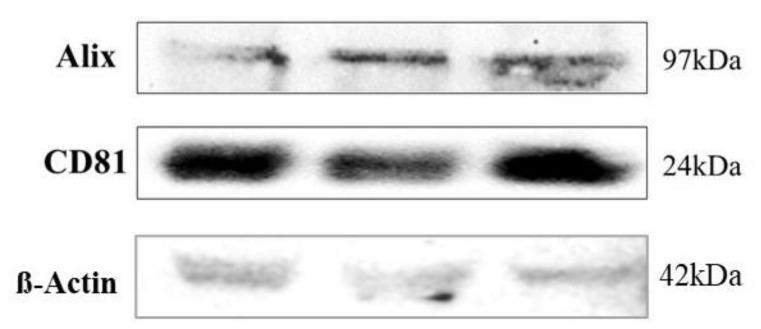
Western Blot results. Characterization of isolated extracellular vesicles by Western Blotting analysis using primary antibodies directed to CD81, Alix and loading control β-actin. Bands were obtained using an exposure of 100 s.

The purity of serum-derived extracellular vesicle preparations was evaluated by western blot analysis, testing for ALIX and CD81 in three random samples (Figure 2). EVs are rich in these two proteins [23].

**Figure 3:**
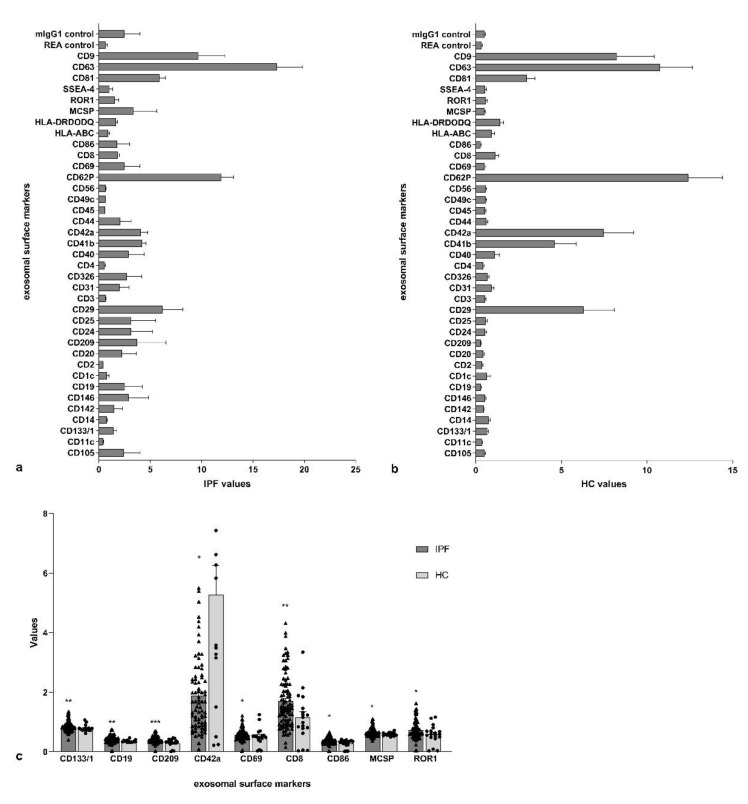
Extracellular vesicle surface marker values and the main differences between the two subgroups. (**a**) Exosomal surface epitope values in idiopathic pulmonary fibrosis (IPF) patients; (**b**) Exosomal surface epitope values in healthy controls (HC); (**c**) the significant differences of exosomal surface epitope values between idiopathic pulmonary fibrosis (IPF) patients and healthy controls (HC). (* *p*: 0.0332; ** *p*: 0.0021; *** *p*: 0.0002).

The exosomal surface epitope values of IPF patients and controls are reported in Figure 3a,b. respectively. CD19, CD69, CD8, and CD86 were significantly higher in IPF patients than in controls (*p* = 0.0023, *p* = 0.0471, *p* = 0.0082, and *p* = 0.0143, respectively) (Figure 3c). CD42a was lower in IPF subjects than in controls (*p* = 0.0153), while CD209, Cd133/1, MCSP, and ROR1 were higher in IPF patients than in controls (*p* = 0.0007, *p* = 0.0050, *p* = 0.0139, and *p* = 0.0335, respectively) (Figure 3c).

**Figure 4:**
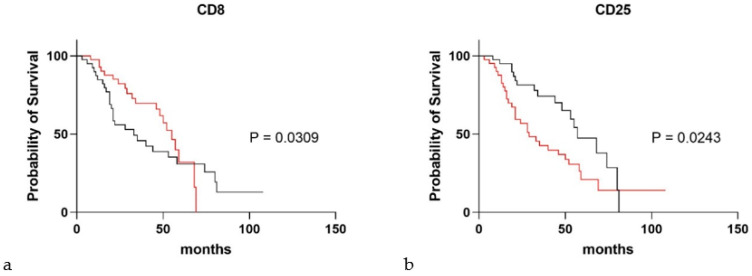
Significant exosomal surface markers in survival analysis of idiopathic pulmonary fibrosis patients. (**a**,**b**) Kaplan-Maier survival analysis dividing idiopathic pulmonary fibrosis patients according to CD8 (cut-off 1.53) and CD25 (cut-off 0.48), respectively. (red), Thres = FALSE; (black), thres = TRUE.

Figure 4 shows the results of Kaplan-Meier survival analysis for IPF patients, stratified according to CD25 and CD8 values. For median values and a cut-off of 0.48 for CD25, the two subgroups showed a significant difference in survival rate (*p* = 0.0243, hazard ratio: 0.52 (95%CI 0.29–0.92) (Figure 4a); the same was true for CD8 (cut-off 1.53, *p* = 0.0309, hazard ratio: 1.39 (95%CI 0.75–2.53) (Figure 4b).

## 4. Discussion

The present multicentre study is the first to examine the expression of 37 exosomal surface epitopes in IPF patients. To our knowledge, no data are currently available in the literature on protein expression in EVs from serum of IPF patients. Only one study has investigated miRNA expression and did so in a murine bleomycin-induced lung fibrosis model and in IPF patients: in the murine model, miR-21-5p in EVs was found to be significantly upregulated in acute inflammatory phase and the later fibrotic phase. This increase in miR-21-5p expression in circulating EVs was also observed in IPF patients and showed a significant correlation with disease progression [4].

Our study identified increased expression of many exosomal surface epitopes (CD19, CD8, CD69, and CD86) in IPF patients with respect to controls, suggesting that an alteration of these markers may be associated with lung fibrosis. From a mechanistic point of view, overexpression of these epitopes in our population is probably related to adaptive immune system dysregulation, which has been described in IPF patients [24,25,26,27]. In particular, CD19 signalling is associated with the development of lung fibrosis through control of B cell infiltration into lung tissue [24]. B cell infiltration is known to be overexpressed and to form lymphocyte aggregates [25]. It was also recently proposed that due to loss of T- and B-cell tolerance, a specific humoral immunity aberration leading to autoimmune responses may be involved in the pathogenesis of IPF, underlining the potential role of these cells [28].

CD69 is a human transmembrane C-type lectin, initially detected on the surface of activated lymphocytes and known as a very early lymphocyte activation marker antigen [29]. It has been demonstrated that CD69 plays an important role in the progression of lung injury induced by bleomycin [30], the typical murine model of IPF. The crosslinking of CD69 induces several cell responses, including nitric oxide production in human monocytes [30], T cell proliferation, production of TNF-α [31], and NK cell cytotoxicity [32]. An increasing trend in the number of CD8 cells expressing the activation marker CD69 has been demonstrated in IPF patients, suggesting possible enhancement in the number of activated T suppressor/cytotoxic cells in IPF [33,34]. CD69 is a useful marker of NK cell cytotoxic activity, whereas proliferative potential is indicated by CD25 expression. CD25 (known as interleukin-2 receptor alpha chain) is important in T cell proliferation, activation-induced cell death, and the activity of regulatory and effector T cells. CD25 is reported to be down-regulated in IPF patients [35,36], and our study revealed low expression of CD25 associated with poor prognosis in IPF patients.

Unfortunately, no data are available on NK-CD69-expressing cells in interstitial lung diseases, although increased NK (CD56+) cells were reported in peripheral blood from IPF patients by Esposito et al. [37]. Interestingly, our study revealed that higher CD8 expression was associated with poor prognosis in IPF patients, confirming results recently reported in the literature. CD86 (also known as B7-2) is expressed by antigen-presenting cells and binds as a ligand to costimulatory CD28 molecules on the surface of all naïve T cells and to the inhibitory receptor CTLA-4 (cytotoxic T-lymphocyte antigen-4) [38]. Kaneko et al. demonstrated that dysregulation of B7 molecules in epithelial cells from IPF patients may lead to activation of autoreactive T-lymphocytes, which contribute to the pathogenesis of fibrosing lung diseases [39]. Regulatory T cells produce CTLA-4 that binds CD86 with higher affinity than CD28: the co-stimulation necessary for proper T-cell activation may therefore also be affected.

Our study showed high CD42a (known as platelet glycoprotein IX (GP9)) expression in EVs from IPF patients. CD42a is expressed on platelets and megakaryocytes [40,41]. Transient activation of platelets induces pro-inflammatory and pro-fibrotic effects [42]. While the role of platelets in IPF is unknown [43,44], platelet trapping in the lungs of mice following intravenous bleomycin administration showed a strong correlation with subsequent collagen deposition, suggesting a role in fibrogenesis in this animal model [42].

CD209 (known as dendritic cell-specific intercellular adhesion molecule 3 (ICAM-3)) was abundantly expressed in our IPF patients. CD209 is a C-type lectin receptor present on the surface of macrophages [45] and dendritic cells. Tsoutso et al. reported normal serum levels of ICAM-3 in IPF patients, unlike those of ICAM-1 and -2 [46]. Bellamri et al. recently demonstrated that nintedanib partially blocks the synthesis of M2a macrophage markers (including CD209), while it does not reduce synthesis of pro-fibrotic cytokines [47].

On the other hand, CD133/1 (known as prominin-1, a pentaspan transmembrane glycoprotein), is a recognized marker of hematopoietic stem cells and committed progenitors [48] and is also expressed on adult epithelial cells [49]. Germano et al. reported that Prominin-1/CD133+ lung epithelial progenitors protected against bleomycin-induced pulmonary fibrosis [50]. In our study, we observed higher expression of CD133/1 in IPF patients than in controls: this result probably reflects the compensation of the profibrotic/antifibrotic protein imbalance typical of IPF [51,52,53,54,55,56,57].

No data are available in the literature on MCSP (melanoma-associated chondroitin sulphate proteoglycan, known as neuron-glial antigen 2 (NG2) or chondroitin sulphate proteoglycan 4 (CSPG4)) in pulmonary fibrosis. MCSP is a surface type I transmembrane core proteoglycan that is crucially involved in cell survival, migration, and angiogenesis [58,59]. However, since IPF pathobiology is characterized by epithelial dysfunction, altered epithelial-mesenchymal transition and consequent reparative processes, the increased expression of MCSP in EVs observed in our study may be related to aberrant neoangiogenesis processes associated with the pathogenetic mechanisms of IPF [60].

The last marker abundantly expressed in EVs of our IPF patients was ROR1 (receptor tyrosine kinase like orphan receptor (1)). This is not surprising since ROR1 and ROR2 are key mediators of WNT5a signalling [61]. WNT5a, a predominantly non-canonical WNT ligand, is increasingly recognized as an important regulator of stem-cell renewal, cell migration, cell polarity, and inflammatory responses and is present in the epithelial and mesenchymal compartments during embryogenesis and mainly in fibroblasts and endothelial cells in adult lungs [62,63]. Vuga et al. showed that WNT5a is a key regulator of fibroblast proliferation and resistance to apoptosis [64], key mechanisms in the development and progression of lung fibrosis in IPF.

Mesenchymal stem-cell-derived EVs have a strong therapeutic effect in cases of organ damage, including acute lung injury [65]. Recent reports outline promising data for future applications of EVs in the treatment of lung diseases. The beneficial effects of EVs are believed to be linked to the transfer of stem cell contents to damaged cells [66]. This unique property qualifies EVs for drug delivery, as novel therapeutic targets and as promising candidates for biomarkers of lung diseases.

This is a novel and intriguing research study contributing to the definition of EVs’ potential role in the pathogenesis of IPF. Although preliminary, the results of this study were validated in two different cohorts of IPF patients recruited from two Italian Regional Referral Centres for ILD. The main limitations of study include the retrospective design of the research, the lack of data from EVs in relation to IPF treatments and the need to compare our results in different biological fluids.

In conclusion, our study shows the expression of surface epitopes on EVs from IPF patients for the first time. It provides interesting data on their communication signatures/exosomal profile in serum from IPF patients, offering new insights into the pathogenesis of the disease. Some of these epitopes proved to reliably predict mid-term survival of IPF patients treated with antifibrotic drugs, suggesting their possible use as prognostic indicators in the management of these patients.

## Figures and Tables

**Table 1:** Main characteristics of our study population including age, gender, smoking habit, BMI, LFT parameters, total protein concentrations (mg/mL, Bradford assay), and antifibrotic treatments started after a certain time.

Parameters	HC (n = 19)	IPF (n = 90)	*p* Value
Age (median *IQR*)	63 (52–65)	71 (66–75)	0.3111
Gender, M/F	12/7	69/21	0.2911
Smoking habit (never/former)	10/9	42/48	0.3102
BMI (kg/m^2^)	26 (24–27)	25 (23–29)	0.0892
Pulmonary function parameters			
FVC %	101.1(88.3–107.2)	68 (59–91)	0.0238
FEV1 %	98 (89.1–110.5)	75 (62–92)	0.0319
DLCO %	89.9 (82.3–98.4)	47 (39–58)	0.0127
EVs total protein concentrations (mg/mL)	2.2 (2–2.3)	2.9 (2.5–3.3)	0.285
Pirfenidone/Nintedanib		53/37	

All data are expressed as the median and IQR. Abbreviations: IPF, idiopathic pulmonary fibrosis; HC, healthy controls.

## Data Availability

The data presented in this study are available on request from the corresponding author.

## Supplementary Materials

The following are available online at https://www.mdpi.com/article/10.3390/cells10051045/s1, Figure S1(a): Cytofluorimetric analysis of exosomal surface epitopes in a representative serum sample from idiopathic pulmonary fibrosis patients analysed using Kaluza software; s1(b): Cytofluorimetric analysis of exosomal surface epitopes in a representative serum sample from healthy controls analyzed using Kaluza software.
