# Supplementary material for: Extracellular Vesicle Surface Signatures in IPF Patients: A Multiplex Bead-Based Flow Cytometry Approach
Source: Cells. 2021 Apr 28;10(5):1045. doi: 10.3390/cells10051045 (PMC8146446; doi:10.3390/cells10051045)
Supplement: Supplementary file 1 [file cells-10-01045-s001.zip › cells-1173143-supplementary.pdf]

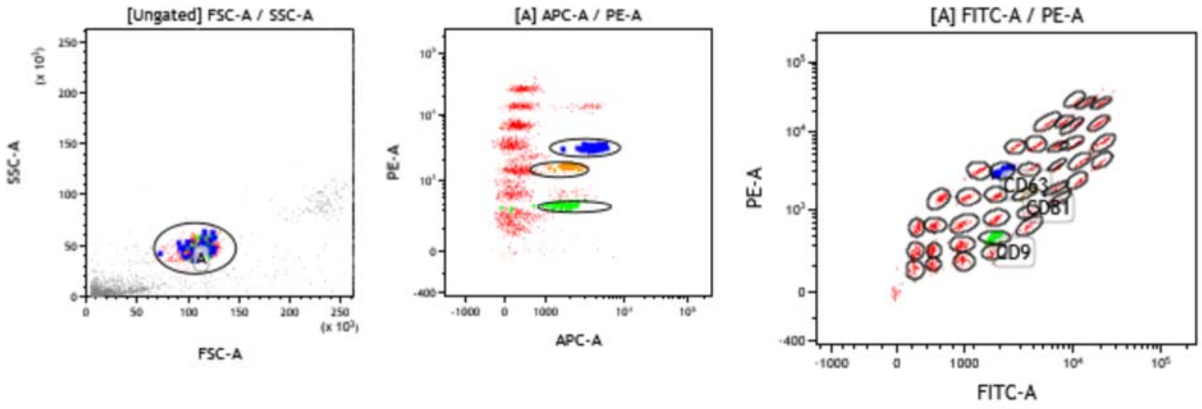

Figure S1a. Cytofluorimetric analysis of exosomal surface epitopes in a representative serum sample from Idiopathic Pulmonary Fibrosis patient analyzed through Kaluza Software.

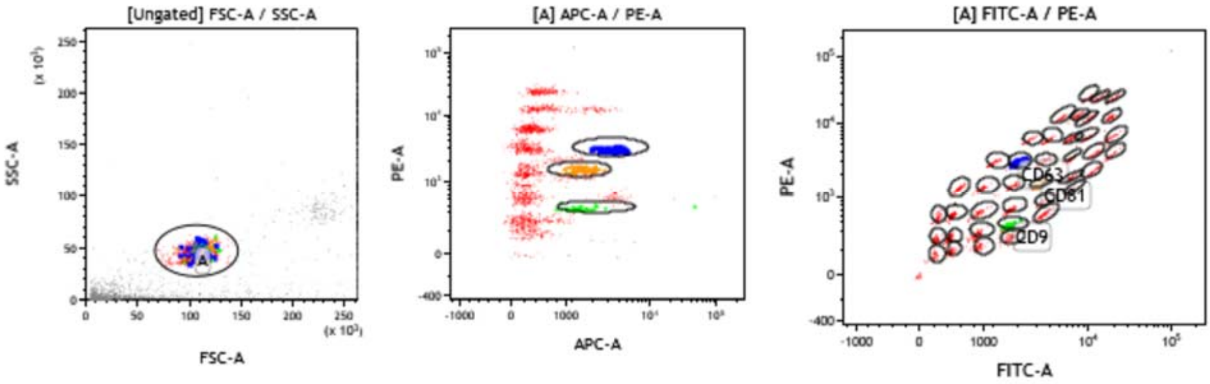

Figure S1b. Cytofluorimetric analysis of exosomal surface epitopes in a representative serum sample from healthy control analyzed through Kaluza Software.
